# Reviews and Book Notices

**Published:** 1882-04

**Authors:** 


### Reviews and Book Notices.

ARTICLE XIII.— A PRACTICAL TREATISE ON IMPOTENCE, STERILITY, AND ALLIED DISORDERS OF THE MALE SEXUAL ORGANS. By SAMUEL W. GROSS, A.M., M.D. Philadelphia: Henry C. Lea's Sons & Co. 1881.

Dr. Gross has grouped together a large number of cases of disease of the male sexual organs, and gives us abundant opportunity to examine the evidence pro and con to substantiate the theories he advances.

In the opening paragraph of the preface, he boldly announces the proposition he endeavors to prove throughout the brochure. He says, concerning impotence and spermatorrhœa: "According to my observations, they usually depend upon reflex disturbances of the genito-spinal center, and are almost invariably induced or maintained by appreciable lesions of the prostatic portion of the urethra, etc." He has observed 137 cases of impotence induced by inflammation and hyperæsthesia of the prostatic portion of the urethra, and twelve cases from diminished or abolished reflex excitability of the genito-spinal center. He claims the latter cases occur in a class of subjects in whom the reflex excitability of the genito-spinal center is induced before prostatic inflammation declares itself, yet in nine of these cases strictures were formed. The question naturally arises, if prostatic inflammation and hyperæsthesia induce impotence, may not prostatic inflammation and subsequent anæsthesia, instead of the genito-spinal centers being impoverished, have produced the impotence in the twelve cases referred to?

The ground that masturbation frequently produces a stricture seems well taken, and proven by abundant authority.

The author divides the book into four chapters: I. Impotence; II. Sterility; III. Spermatorrhœa; IV. Prostatorrhœa. Impotence he subdivides into atomic, psychical, symptomatic and organic. Sterility, he argues, is due to azoöspermism, aspermatism and misemission. There are sixteen illustrations.

J. A. R.

Article XIV.—Lectures upon Diseases of the Rectum and the Surgery of the Lower Bowel. By W. H. Van Buren, M.D., LL.D., Professor of the Principles and Practice of Surgery in the Bellevue Hospital Medical College. New York: D. Appleton & Co. 1881.

This work is the second edition, considerably improved and enlarged over the previous edition. It certainly deserves the place of being the most recent and intelligent work we have on the diseases of the rectum and their treatment.

J. A. R.

Article XV.—The Nature and Treatment of Rabies or Hydrophobia. Second Edition. By Thomas M. Dolan, L.R.C.P., etc., Physician to the Halifax Hospital.

An elaborate collection of the literature of the subject, with an hundred and fifty cases detailed as to clinical history and post-mortem examination. The results of various modes of treatment are compared. Modification of the disease by temperament and physical condition receives much attention.

The little book is a valuable one for reference in the history of the subject, and a useful one to the busy physician.

Article XVI.—Posological Table, Including all the Officinal, and most frequently employed Unofficinal Preparations. By Charles Rice, Chemist, Department of Public Charities and Corrections. N. Y.: William Wood & Co.

A handy little book for the prescription counter or the pocket. The doses given in the usual apathecaries' system are easily converted to the metric by tables appended.

Article XVII.—Ophthalmic and Otic Memoranda. By D. B. St. John Roosa, m.d., and Edward T. Ely, m.d. Revised Edition.

This little work aims to give but a bare outline of the subject of which it treats, a sort of dictionary of ophthalmic and aural science. As such it is very good.

Article XVIII.—A New Form of Nervous Disease, together with an Essay on Erythroxylon Coca. By W. S. Searle, a. m. m.d.

The author describes at length a nervous disease which he has been unable to classify, characterized by a sensation of sudden shock, or blow, or explosion in some part of the head, usually the occipital region, and sometimes preceded by something similar to the aura of epilepsy, almost uniformly accompanied by intense vertigo, without convulsions or frothing at the mouth, or dilated pupils. No drowsiness or mania follows the attack ; loss of consciousness rarely occurs. The author quotes from Hammond, Nothmayl and others that it is not epilepsy nor epileptiform, and denies it to be Beard's neurasthenia.

The essay on erythroxylon coca is very complete and interesting, the author believing that for nerve waste and excessive fatigue this is the remedy *par excellence.*

Article XIX.—The Student's Dose Book and Anatomist Combined. By C. Henri Leonard, a.m., m.d., Professor, Medical and Surgical Diseases of Women, Michigan College of Medicine.

The most complete and compact collection of things useful to the physician, surgeon or student, we have seen.

Article XX.—Sea Air and Sea Bathing. By John H. Packard, Surgeon to the Episcopal Hospital, etc.

This little manual for those who resort to the sea-shore, is comprised in ten short, attractive chapters, on general considerations of seaside resorts: bathing in the sea; accidents in bathing; sea bathing for invalids; amusements at the sea shore; cottage life; sanitary matters; the sea-shore as a winter resort, and excursions to the sea shore. P. Blakiston, publisher.

ARTICLE XXI.—THE SCIENCE AND ART OF MIDWIFERY. By WILLIAM THOMPSON LUSK, A.M., M.D., Professor of Obstetrics and the Diseases of Women and Children in the Bellevue Medical College, etc., etc. With numerous Illustrations. 8vo. cloth, pp. 687. New York: D. Appleton & Co. 1882.

One of the best treatises on obstetrics, finely illustrated, and issued in the accustomed style of the books published by the Appletons. It is one of the first works on the subject by American authors, and should on that account become the accepted text-book in most of our colleges. Of course such a work cannot exhibit a great deal of originality; the one under consideration is especially remarkable for its well-sifted literature, appropriately chosen illustrations, and its scientific data above all. It is also better adapted to this country than any other.

ARTICLE XXII.—THE SYMPATHETIC DISEASES OF THE EYE.—By LUDWIG MAUTHNER, M. D. Translated from the German by Warren Webster, M. D., and James A. Spalding, M. D. New York, William Wood & Co. 1881.

We fully agree with the translators in saying that there is an urgent necessity for general practitioners to have a clear and reliable description of the multiform symptoms, and the treatment of sympathetic ophthalmia, so that they may at once recognize its presence, and act accordingly. But we seriously doubt whether Mauthner has succeeded in furnishing to the profession the desired book.

Books for the busy practitioner must impart the greatest amount of information in the fewest possible words; concise language and brevity are their greatest merits, prolixity of style is their worst fault. But that is just the fault we find in all of Mauthner's writings, that he often fails by the multitudes of words to convey clear ideas to the reader.

So in his present book, though it is the best, completest compilation ever published on sympathetic diseases of the eye, and highly interesting for the specialist, the statements of facts and the descriptions are so often interpolated by theoretical digressions that its value as a ready reference book for the busy practitioner is seriously impaired.

ARTICLE XXIII.—A TREATISE ON THE DISEASES OF THE EYE, By HENRY D. NOYES, A. M., M. D., Professor of Ophthalmology and Otology in Bellevue Medical College; Surgeon to the New York Eye and Ear Infirmary, etc. New York, William Wood & Co., series 1881.

The difference in the styles of the German and American medical authors could not be better illustrated than it is in Mauthner's and Noyes' books. Both authors set out for the same goal of writing a book for the instruction of practicing physicians. Although their works are of a different scope, the manner in which they acquitted themselves of their respective tasks, is very characteristic. Mauthner, selecting one single subject, spins out his lecture by prolixity and theoretical dissertations to a book of considerable size. Noyes, going over the whole field of ophthalmology, was so fearful lest his book should exceed the prescribed limits and convenient size, that he excluded religiously all superfluous discussions and everything non-essential to his practical end. And we fully agree with his view that a text-book for students and practitioners is not the proper ground for discussing the pros and cons of open questions and new theories.

The reader will find Noyes' book remarkably free from such digressions, and, therefore, derive more pleasure and benefit from its perusal than he is accustomed to receiving from ophthalmic works. The author's style is exceedingly lucid and concise, his views upon the merits or demerits of medical or surgical treatment, as the case may be, are couched in such definite language that there can be no doubt about his position.

There are, of course, some imperfections in the work, (for what book could be pronounced absolutely faultless?), which, however, with one or two exceptions, are, perhaps, only a matter of judgment. Possibly, we attach greater value to the practical importance of certain affections, than the author was disposed to do. For instance, we failed to find any reference to seborrhœa of the tarsal borders, which disorder is often so annoying, and the occasional cause for such loud complaints on the part of the patient, that it should be considered in a text book. We also should have given that peculiar phlyctenular inflammation of the cornea, known as fascicular keralitis, greater prominence, and we should most decidedly have incorporated in a treatise for practitioners Dr. Kipp's valuable description of malarial keratitis. But, as we said before, these omissions may be accounted for by a difference of judgment, and they do not mar the general excellence of the book.

A strange mistake, however, occurs in the chapter on entropium. The text describing the operation for this deformity and the fig. 67, illustrating the procedure, are at war with each other, the illustration picturing one method and the text being a descriptive compound of two operative procedures which widely differ from each other in all essential features.

We mention these defects, not because we wish to be hypercritical, or to detract anything from the value of the work, but simply for the purpose of bringing them to the notice of the author, and we hope he will soon have an opportunity of eliminating these blemishes in a new edition of his otherwise excellent text-book. F. C. H.

### ARTICLE XXIV.—ILLINOIS STATE MEDICAL SOCIETY—TRANSACTIONS. 1881.

A pretty octavo volume of 362 pages, bound in cloth, and beautifully printed. It contains many valuable papers, several of them remarkable for their originality and practical value; such, for instance, is the article of Dr. E. Fletcher Ingals on "Laryngeal Tumors." Some of the reports, as that on "Ophthalmology and Otology," by Dr. F. C. Hotz, are almost perfect in their way.

"Dysmenorrhœa," by Dr. Ellen A. Ingersoll, contains many different views from those long accepted, yet her hints are so plausible, and well sustained by her own practical experience, that her paper will remain one of the best, and one which should be often referred to.

"The Present Status of Specialism in Medical and Surgical Practice," a spicy criticism by Dr. A. Reeves Jackson, enlivened its auditors at the annual meeting, and is certainly the most readable of any contribution to the present Transactions.

It would be difficult to qualify each article in the appropriate manner, for they are all worthy of praise, and they furnish to the busy practitioner an epitome almost of what is at present known in the various departments of medicine and surgery.

ARTICLE XXV.—GENERAL MEDICAL CHEMISTRY, FOR THE USE OF PRACTITIONERS OF MEDICINE. By R. A. WITTHAUS, A.M., M.D., Professor of Chemistry and Toxicology in the Medical Department of the University of Vermont; Professor of Physiological Chemistry in the Medical Department of the University of the City of New York; Member of the Chemical Societies of Paris and Berlin, etc. etc. 8vo, cloth, pp. 443. New York: William Wood & Co. 1881. (Wood's Medical Library.)

This is perhaps the best book in the series of 1881, and is unique in more than one respect. It supplies a want long felt by the medical profession in this country, and is so complete as to be as useful to chemists and professors of chemistry as to ordinary students.

The various organic compounds are treated of at greater length than in many text-books, forming, under the name Compounds of Carbon, more than half the present volume, which is thereby likely to help the manufacturing chemist and the toxicologist to a great extent. Chemical philosopy is treated of in a masterly manner, and the same can be said of the tests and reactions of each element.

In fact, this book does honor to a Library of Standard Medical Authors, and will induce some, doubtless, to buy the whole series, in order to possess a good text-book of medical chemistry.

ARTICLE XXVI.—ILLUSTRATIONS OF DISSECTIONS, in a Series of Original Colored Plates, the Size of Life, Representing the Dissection of the Human Body. By GEORGE VINER ELLIS, Professor of Anatomy in University College, London, and G. H. FORD, Esq. (Reduced on a uniform scale.) Vol. I, Second Edition. 8vo, cloth, pp. 233. New York: William Wood & Co. 1882. Wood's Library of Standard Medical Authors.

It seems that in issuing the present volume, the publishers desired to improve yet on former series. The plates, twenty-eight in number, are beautiful lithographs, and very accurate. The coloring is as well executed as we should expect in a work of this character, and the descriptions are full and accurate. One cannot easily understand how such valuable works can be given away at such low prices, and few practitioners now can afford to be without Wood's Library.

ARTICLE XXVII.—ESSENTIALS OF THE PRINCIPLES AND PRACTICE OF MEDICINE. By HENRY HARTSHORNE, A.M., M.D., Editor of American Edition of "Reynold's System of Medicine," etc. Fifth Edition, thoroughly Revised and Improved, with 144 Illustrations. 12mo. cloth, pp. 669. Philadelphia: Henry C. Lea's Son & Co. 1881. Chicago: Jansen, McClurg & Co., 117 and 119 State Street.

Probably no book on the Practice of Medicine has ever enjoyed the popularity of this one. A small book, or rather a monograph, a few years ago, it has rapidly evolved into a respectable handbook, which the reviewer has met on the table of every busy physician, and their verdict always has been: An indispensable book. No work ever exhibited a better average of actual practical treatment than this one; and probably not one writer in our day had a better opportunity than Dr. Hartshorne for condensing all the views of eminent practitioners in a 12 mo. The numerous illustrations will be very useful to students especially. These essentials, as the name suggests, are not intended to supersede the text-books of Flint and Bartholow, but they are the most valuable in affording the means to see at a glance the whole literature of any disease, and the most valuable treatment.

The 377 prescriptions at the end of the volume are very well selected, but we hope the next edition will have them translated into the Metric System at the same time with the old, as this lack is the sole defect of the entire work.

ARTICLE XXVIII.—A MANUAL OF HISTOLOGY, Edited and Prepared by THOMAS E. SATTERTHWAITE, M. D., of New York, in association with Drs. THOMAS DWIGHT, J. COLLINS WARREN, WILLIAM F. WHITNEY, CLARENCE J. BLAKE, and C. H. WILLIAMS, of Boston; J. HENRY C. SIMES, of Philadelphia; BENJAMIN F. WESTBROOK, of Brooklyn; EDMUND C. WENDT, ABRAHAM MAYER, R. W. AUNDON, A. R. ROBINSON, W. R. BIRDSALL, D. BRYSON DELAVAN, C. L. DANA, and W. H. PORTER, of New York City. With 198 Illustrations; 8vo., cloth, pp. 478. New York: William Wood & Co. 1881.

This is a carefully prepared volume, likely to meet the demand for such works, which, of late, are becoming rather numerous. However, a little economy in *blanks* and *interlines* had been to the advantage of the buyer. Histology is the most neglected, and the least practical branch of medicine, besides involving unprofitable expenses; yet the returns have not been fruitless in late years, and it is quite desirable that the present generation of students should pay some attention to it, for it is generally in the new fields that discoveries are made. As a pastime, none, perhaps, can compare with the use of a good microscope in the hands of a physician of some learning. Within a few years, microscopes have become cheaper and cheaper, so that good home-manufactured instruments may now be bought for a hundred dollars, which is yet too dear a luxury for most students, though indispensable to the practitioner. We would recommend the "Manual of Histology" to any physician or student who desires to be proficient in the medical sciences.

ARTICLE XIX.—THE HEART AND ITS FUNCTION forms one of the series of Health Primers published by D. Appleton & Co., and like the others, is brief, simple, and elementary in statement, suitable for the guidance of intelligent adults.

ARTICLE XXX.—A SYSTEM OF SURGERY, THEORETICAL AND PRACTICAL. In Treatises by Various Authors. Edited by J. HOLMES, M. A. First American, from Second English Edition, Thoroughly Revised and Much Enlarged by JOHN H. PACKARD, A. M., M. D., Assisted by a large corps of the most Eminent American Surgeons. In Three Volumes, with many Illustrations. Vol. II, 8vo., half Russia, pp. 1063. Philadelphia: H. C. Lea's, Son & Co. 1881.

The present volume, issued soon after the first, treats of Diseases of the Eye, Ear, Nose, Tongue, the Circulatory System; the Absorbent System; the Mouth, Teeth, Intestines, Rectum; Hermia; Diseases of the Genito-Urinary Organs; Surgical Diseases of Women. This last is by Jonathan Hutchinson. Among the American revisers are J. Solis Cohen, Lewis A. Stinson, James Truman, D. D. S., J. H. C. Simes, John H. Packard, Edward L. Keyes, etc. The illustrations are 309 in number, and four lithographs. The second is not inferior to the first volume, and deserves the patronage of the profession, the world over. The rapid specializing of the various branches of surgery, together with their enormous increase in our day, have rendered it impossible henceforth for any surgeon to write an intelligent text-book on the subject, so that the book here presented is indispensable for ordinary physicians as much as for specialists, and all those practitioners who are made to feel the pressing necessity of keeping up with the rapid advance in surgery. We wish the proof-reader to be more careful in his treatment of the last volume.

H. D. V.

---

THE Woman's Medical College of Baltimore has recently been incorporated. The course of lectures will begin October 1, 1882. Seven professors, all of them gentlemen, have already been appointed.

